# Latest Research of Doped Hydroxyapatite for Bone Tissue Engineering

**DOI:** 10.3390/ijms241713157

**Published:** 2023-08-24

**Authors:** Diana-Elena Radulescu, Otilia Ruxandra Vasile, Ecaterina Andronescu, Anton Ficai

**Affiliations:** 1 Department of Science and Engineering of Oxide Materials and Nanomaterials, Faculty of Chemical Engineering and Biotechnologies, Bucharest National Polytechnic University of Science and Technology, 011061 Bucharest, Romania; radulescu.diana95@gmail.com (D.-E.R.); otilia.vasile@upb.ro (O.R.V.); anton.ficai@upb.ro (A.F.); 2 National Research Center for Micro and Nanomaterials, Bucharest National Polytechnic University of Science and Technology, 060042 Bucharest, Romania; 3 Romanian Academy of Scientists, 050045 Bucharest, Romania

**Keywords:** hydroxyapatite, doping, bone tissue engineering, biocompatibility, mechanical properties

## Abstract

Bone tissue engineering has attracted great interest in the last few years, as the frequency of tissue-damaging or degenerative diseases has increased exponentially. To obtain an ideal treatment solution, researchers have focused on the development of optimum biomaterials to be applied for the enhancement of bioactivity and the regeneration process, which are necessary to support the proper healing process of osseous tissues. In this regard, hydroxyapatite (HA) has been the most widely used material in the biomedical field due to its great biocompatibility and similarity with the native apatite from the human bone. However, HA still presents some deficiencies related to its mechanical properties, which are essential for HA to be applied in load-bearing applications. Bioactivity is another vital property of HA and is necessary to further improve regeneration and antibacterial activity. These drawbacks can be solved by doping the material with trace elements, adapting the properties of the material, and, finally, sustaining bone regeneration without the occurrence of implant failure. Considering these aspects, in this review, we have presented some general information about HA properties, synthesis methods, applications, and the necessity for the addition of doping ions into its structure. Also, we have presented their influence on the properties of HA, as well as the latest applications of doped materials in the biomedical field.

## 1. Introduction

**Figure 1:**
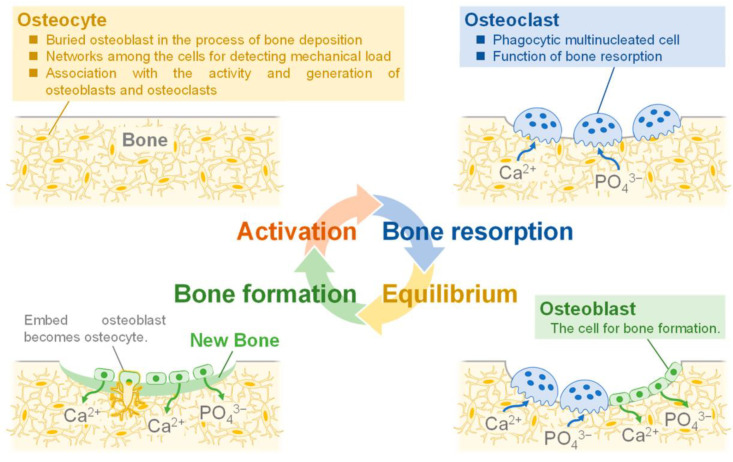
Representation of bone formation process [3].

Over the past years, interest in the development and application of biomaterials for bone tissue engineering has increased extraordinarily. Since bone regeneration is considered the most complex and well-orchestrated physiological process, the main purpose of researchers has been to design biocompatible materials with well-defined biomechanical and physicochemical properties that can enhance bone formation and regeneration (Figure 1). Further, these biomaterials aim to initiate specific cellular responses at a molecular level, from cell interaction, proliferation, cell attachment, and differentiation to extracellular matrix synthesis [1,2]. 

Moreover, biomaterials are a distinct class of materials extensively used in bone tissue engineering. They have been comprehensively studied and applied in the biomedical field due to their similar microstructure with the osseous tissue and biologic-responsive properties, such as biocompatibility, osteoconductivity, osteoinductivity, osseointegration, and superior mechanical properties. Although they present numerous advantages, many drawbacks are still present, such as wear, corrosion, the release of toxic ions, stress-shielding limitations, and, in some cases, the necessity for the removal of non-degradable materials. To surpass these limitations, novel materials have been investigated, in which hydroxyapatite (HA) has been widely applied [4,5].

Thus, HA has been extensively studied by researchers in biomedical applications due to its osteogenic properties, biocompatibility, capacity to form a strong bond with the osseous tissues, and natural occurrence in the human body. This material has been mainly used in bone-healing applications. Even though synthetic HA presents excellent results in bone tissue engineering, the material has poor mechanical strength, low fracture toughness, and brittleness, which marks it unsuitable for load-carrying applications [6]. To surpass these disadvantages, several methods have been studied to increase its physicochemical and biological properties, mainly the osteoconductivity of synthesized HA. Hence, the optimum solution to obtain this goal was to alter the structure of HA by doping metal or non-metal ions, or even their composites. The inclusion of nanoscopic trace elements into HA can lead to modifications in crystallinity, lattice, and dispersal kinetics and improve mechanical properties and biological activity [7,8]. 

Another essential drawback of the applications of HA in bone tissue engineering is the presence of infections generated by pathogenic microorganisms. These microorganisms can easily infect the implantation site or the implant itself during surgical intervention. This issue may lead to increased treatment costs, as additional surgeries may be needed, or the rehabilitation time may be extended [9]. Moreover, the use of antibiotics does not ensure sufficient protection against bacterial strains resistant to multiple antibiotics. Hence, the doping of HA with different cations including zinc (Zn), silver (Ag), cerium (Ce), strontium (Sr), magnesium (Mg), copper (Cu), or titanium (Ti) ions is the ideal solution to enhance the antimicrobial activity at the implanted site, as they are not cytotoxic at low concentrations [10]. 

**Figure 2:**
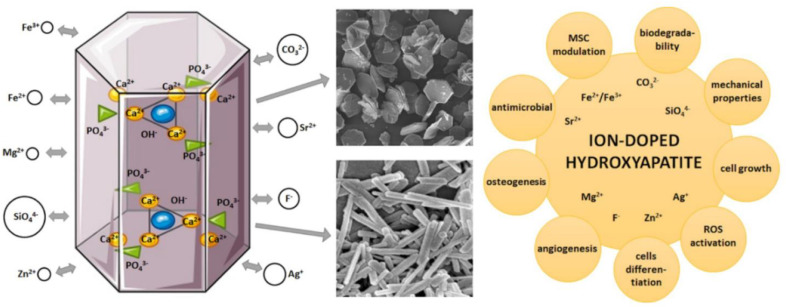
Representation of HA crystal structure and applications of doped nanoparticles [12].

The atomic substitution or doping of HA (Figure 2) is the best approach to also improve the main weaknesses of implants based on HA, mainly the mechanical properties. By performing different types of substitutions, especially anionic (PO_4_^3−^) and cationic (Ca^2+^) substitutions, all the drawbacks mentioned before can be solved [11].

In this regard, Bassyouni et al. reported that, by increasing the degree of crystallinity of HA and decreasing its grain size, the bioavailability of the material would be improved. Also, for the increase in dissolution rate, appropriate structural changes could be considered. The structure of HA can be modified by:

- replacing the Ca^2+^ sites with Zn^2+^, Sr^2+^, Mg^2+^, and Na^+^ ions;
- replacing the PO_4_^3−^ sites with CO_3_^2−^, or SiO_3_^2−^ ions;
- replacing the −OH group with Cl^−^, F^−^, and CO_3_^2−^ ions.

These examples of suggested ionic exchanges lead to the disturbance of crystallinity, surface charge, lattice parameters, and morphology, which can determine variations in the thermal stability, mechanic properties, solubility, and bioactivity of HA [13].

This review aims to present the main features of HA in the medical field, including their properties and synthesis methods, but also to present the importance of functionalization by doping HA. Moreover, we have summarized the influence of doping materials and the latest applications in biomedical engineering.

## 2. General Aspects of Hydroxyapatite Applied in Bone Tissue Engineering

### 2.1. Physical and Mechanical Properties

**Figure 3:**
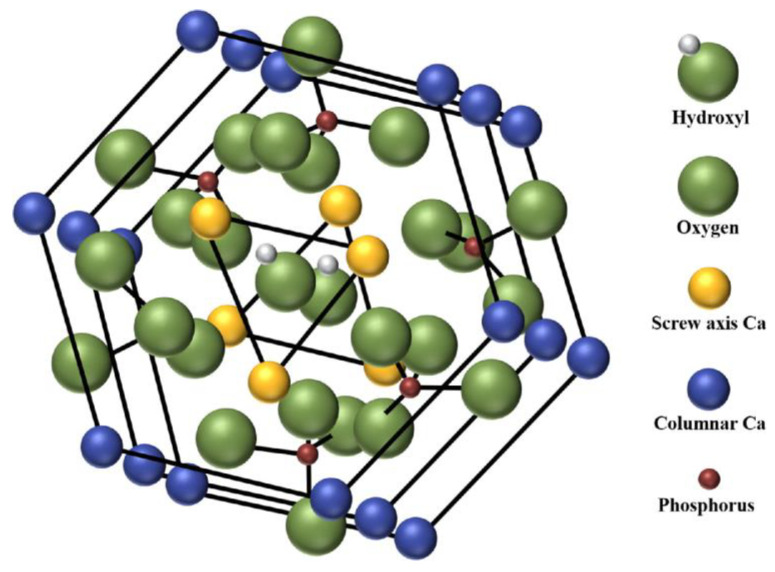
The crystal structure of HA [16].

Ca_10_(PO_4_)_6_(OH)_2_ (HA) is part of the calcium phosphate (CP) ceramic group found in human osseous tissue bone (around 60–65%), a natural mineral compound. This material has been preferred to be applied as bone graft material due to its resemblance with the composition of the native hard tissue, with a stoichiometrically similar Ca/P ratio (1.67). Further, it is also well known for its good osteoconductivity, biocompatibility, and bioactivity. Nevertheless, synthesized HA (Figure 3) does not possess the specific trace ions which are normally found in the chemical structure of the osseous tissue that aids in bone formation, and, therefore, it possesses inferior bone regeneration [14,15]. 

The high purity and variable stoichiometry of HA have led to increased interest in its use in biomedical applications, mainly in bone tissue engineering. These materials, especially nanoparticles, present improved densification and sintering capabilities due to their high surface energy. Moreover, its enhanced wear resistance, chemical stability, and similar composition to the native tissue make this biomaterial more suitable compared to polymers or metals [17]. HA powder can be obtained through chemical synthesis routes at temperatures ranging between 60 and 85 °C by using precursors containing phosphate and calcium. The −OH groups increase the absorbance capacity for biomacromolecules and dielectric properties. Moreover, it increases osseointegration and bioactivity [18,19]. Also, its intrinsic osteoinduction, osteoconduction, and osteogenic properties are ideal for its use in the biomedical domain. Still, the use of synthetic HA is, at present, limited because of the insufficient mechanical and morphological limitations connected to cell interaction and infiltration [20].

Generally, the mechanical properties of the biomaterials applied in bone tissue engineering relate to their specific surface, pore size, pore distribution, total pore volume, and porosity. In addition, a ceramic with high porosity, inhomogeneous pore size distribution, superior total pore volume, large pore size, and large specific surface area will present low strength. Moreover, the bulk moduli and elastic constants of HA are mechanical properties that directly influence the strength of HA implants or scaffolds. For example, the porosity decreases the mechanical strength of the ceramic, thus reducing the cross-sectional area when a load is applied. Therefore, reducing the porosity, pore size, specific surface area, and total pore volume will improve the strength of the ceramic [21,22,23]. 

Essentially, the presence of porosity on the surface of the implant increases the depth, which corresponds to nano-HA accuracy in reducing calcium deficiency and surface roughness. This development is vital to the healing process. Moreover, increasing the HA microporosity may lead to an increase in the surface area in direct contact with body fluid, thus accelerating biodegradation and enhancing the deposition of the apatite on the surface of the material, improving the bioactivity of the material. By taking into consideration the aspect mentioned above, a proper balance between the porosity of the material and its mechanical properties is necessary [24,25].

The main requirement in the successful application of HA in bone tissue engineering is to provide sufficient mechanical strength until completed regeneration and new tissue formation, to offer suitable support. Furthermore, HA mechanical properties obtained by sintering are directly influenced by pore shape, material density, pore connection, and grain size. All these parameters can be attuned by the sintering temperature. In this direction, Trzaskowska et al. mentioned that, by increasing the sintering temperature, the compressive strength and density of HA are improved; meanwhile, the porosity and elastic deformation, according to Young’s modulus values, decrease [24]. Additionally, Benaqqa et al. reported that the application of HA sintered at high temperatures is not allowed, especially due to high brittleness. The obtained HA is very susceptible to the generation of small defects, slow crack growth, and, finally, eventual ceramic failure. Moreover, a sintering temperature higher than 900 °C led to the formation of large grains, reduced porosity, high crystallinity, low specific surface area, and high biocompatibility but is limited in clinical applications [26]. Although the main approach to obtaining HA is based on high sintering temperatures and high-pressure compaction, the obtained material has high porosity and loses uniformity with the generation of cracks. Mechanical properties are altered while the hydroxyl functional groups in HA are eliminated and the material is decomposed to α-tricalcium phosphate (α-TCP), β-tricalcium phosphate (β-TCP), and tetra calcium phosphate (TTCP); but, most importantly, the biocompatibility decreases [27].

Another important property of the material is represented by tensile strength. At the interaction with the osseous tissue, tensile forces are developed at the interface between the material and the tissue by the difference in the modulus of bone-material elasticity. HA fracture toughness also limits the applications of the material, as the required values need to be similar to the ones of the native tissue [28].

Crystallization is another property that has a great influence on the use of HA. While crystallization increases, the abrasion is decreased and reinforces a higher molecular weight of HA. This results in an improved tensile strength as the nanometer size of the bone improves. The extent to which the scaffold mimics the native osseous tissue also influences the crystallite size, increasing the abrasion resistance and reinforcing in vivo loading [25]. Defects such as dislocation and their density may also influence the bioactivity of HA crystals. The dislocations impact the processes that lead to bone apposition on bioactive materials. Moreover, dislocations also are a highly important factor in vivo dissolution processes and in the crystal maturation of biological cells/tissues. Additionally, the dislocations allow plastic deformations through slips. Researchers have proved that the hexagonal lattice of HA crystal shows highly anisotropic plastic deformation at increased temperatures [29,30].

On the other hand, the bioactivity of the material has also been thoroughly considered. This characteristic is represented by the capacity of the material to directly “bond” to the osseous tissue through chemical interactions. Commonly, when HA is implanted into the human body, a fiber-free and carbonated apatite with fiber-free layers is formed on the surface, improving the bonding and the fixation with the surrounding tissue [31]. The properties of the material surface are influenced by both chemical and topological aspects. The surface chemistry controls the dispersion of all charges and electric dipole moments at the interface between the material and tissue, generating electrostatic interactions in the aqueous environment with the proteins. In this regard, several factors must be considered when the surface of the material is modified. One of these factors is represented by the hydrophilicity of the surfaces, which promotes cellular adhesion [32].

Siddiqui et al. [33] demonstrated that the electrostatic interactions of the HA surface, with Ca^2+^ and PO_4_^3−^ ions encourage bone-like apatite formation. They reported that, when the HA surface is exposed to simulated body fluid (SBF), the surface displays a negative charge and interacts with the Ca^2+^ ions. In this process, an amorphous calcium phosphate layer rich in calcium is formed, altering the charge to become positively charged. The process continues with the electrostatic interaction with PO_4_^3−^ ions and leads to the formation of amorphous calcium phosphate that is deficient in calcium, which eventually matures into bone-like apatite.

Ultimately, HA presents poor fracture toughness, brittleness, undesirable tribological response, and low hardness. All recent research findings mentioned that, even though the chemical characteristics are similar, there is still a significant difference between the properties of osseous tissue and synthetic HA [34,35]. Nonetheless, the best feature of HA is represented by the capacity to take cationic and anionic substituents, improving their properties for numerous applications. In this regard, the properties of HA must be tailored in such a manner that it should improve cell proliferation and hinder prosthetic device failure for in vivo applications [36]. 

### 2.2. Synthesis Methods

**Scheme 1:**
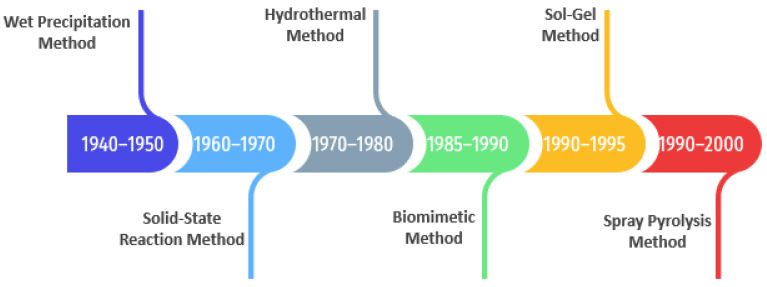
Chronological representation of HA synthesis routes [40,41,42,43,44,45,46,47,48,49,50].

It is well known that both synthetic and natural HA present superior biocompatibility, osteoconductivity, chemical support, and bioactivity, which aids bone tissue regeneration. These are the fundamental characteristics of scaffold applications or coatings used in tissue engineering [37]. Synthetic HA can be obtained through two principal methods: top-down and bottom-up approaches. The top-down method involves the successive cutting or slicing of bulk materials to obtain nanoparticles. On the other hand, the bottom-up method is represented by the build-up of material from the bottom, atom by atom, molecule by molecule, or cluster by cluster. The majority of the published research findings have been focused on the bottom-up method to obtain HA [38]. An abundance of reports has focused on the synthesis of HA powders through solid-state reactions, hydrothermal methods, sol–gel processes, microemulsion, emulsion, and also chemical precipitation. All these synthesis methods have been largely investigated by numerous researchers over many decades, as shown in Scheme 1, to improve their efficiency and functionality. These techniques have been preferred due to their simplicity and cost-effectiveness. Still, the obtained material might lack several essential ions, for example, Na, Sr, Mg, Si, K, and Fe [39].

The conventional solid-state technique is the preferred method to synthesize HA, as the developed nanoparticles preserve the morphology of the phosphate precursor particles. Alternatively, the chemical precipitation method is performed using precursor materials and an aqueous solution at low reaction temperatures, leading to the development of porous nanoparticles. The precipitation method is another chosen method for synthesizing nanosized HA with high purity due to its simplicity and low costs. The properties of the obtained HA depend on the technique used for synthesis, the selected precursors, and the processing parameters (reactant addition rate during the reaction, reaction temperature, and aging time) [51]. These aspects could affect their crystallinity, size, and shape (Table 1).

Another highly studied synthesis method is represented by hydrothermal synthesis. During this technique, the interaction is between the phosphate solution and the calcium precursor in the presence of water, an organic solvent, or a mixture of aqueous/organic. The reactions occur under the well-defined parameters of temperature or high pressure, which can be compared to those under autoclave pressure. Further, this technique generates chemical structures and develops nuclei that form HA with superior crystallinity [68]. Mushtaq et al. [69] mentioned that this technique is not only cost-effective and environmentally friendly, but that it can also easily vary in particle morphologies and sizes, which is a great advantage for biomedical applications. Likewise, the technique can be combined with additional methods, such as microwaves, hot-pressing, ultrasound, mechanochemistry, or electrochemistry. Moreover, Indira et al. [70] suggested that they can facilitate the synthesis time. For example, microwave irradiation efficiently increased the temperature and reaction kinetics compared to conventional methods. On the other side, microwave application leads to good homogeneity and increased fine particle purity.

Between those techniques, the sol–gel method presents many advantages, such as high purity, low synthesis temperature, excellent chemical homogeneity, and good mixing of the starting materials. The synthesis of HA using sol–gel demands that the Ca/P ratio is 1.67. However, depending on the experimental conditions (pH, temperature, aging time), different quantities of β-TCP can be formed [71]. Nellis et al. also confirmed that this route offers the capacity to obtain more versatile materials with good biocompatibility [72]. Furthermore, Ahmadi et al. mentioned that the sol–gel dip-coating approach is based on mixing phosphorus and calcium precursors. This technique was highlighted due to its advantages such as low cost, process temperature, accurate control of chemical composition, higher biocompatibility, and coating thickness. In the sol–gel process, numerous parameters (diluent type of HA solution, type of phosphorus and calcium precursors, temperature, calcination temperature, aging time) and the formation of inter-layer and residual stress in the coating have a substantial influence on the uniformity and quality of the coating [73].

Co-precipitation is another method that is highly studied in HA synthesis. This synthesis pathway aids the formation of the different morphologies of the particles with a low-crystalline nature [74]. Additionally, wet chemical precipitation represents one of the most used techniques due to its simplicity, cost, and low reaction temperature, in which high ratios of pure product can be obtained. As mentioned by numerous researchers, this technique depends on the mechanism of growth–agglomeration–nucleation–aggregation–agglomeration. The procedure beings with the preparation of alkaline (Ca source) and acid (P source) solutions, continued by the reaction between them. Next, there are the stages of precipitation, filtration, drying, and, finally, heat treatment, respectively [75]. The obtained materials through this method were proved to increase bone regeneration, surface absorption, and mechanical properties. On the other hand, more efficient synthesis methods with simplified preparation procedures and short duration are needed [76]. 

Nevertheless, conventional synthesis methods may generate toxicity and environmental damage, mainly air and water pollution, due to the usage of toxic solvents and harsh chemicals. Moreover, researchers have concluded that conventionally synthesized HA does not possess substantial biological properties. Considering this, eco-friendly synthesis methods of HA from natural resources (eggshells, fish bone, seashells, and cow bone) have been successfully developed due to their availability, nontoxicity, and lower cost. The extraction and purification of HA have been further studied in recent years to enhance their quality and expand the application domains [77,78,79]. Another advantage of this route is the capacity to retain some beneficial trace elements from the natural source of the synthesized material. Furthermore, mechanical and thermal processing can be also improved [80,81]. 

Studies on the naturally derived HA have indicated that these preparation methods presented trace elements such as Na, Mg, Sr, and K, but also carbonate anions, which are essential for bioactivity enhancement. Furko et al. mentioned that the most investigated preparation method is calcination, which can lead to obtaining HA powders with superior crystallinity in a fast and economical production process that requires less chemical consumption [82]. To obtain natural HA from eggshells, fish bones, or bovine bones, several chemical and physical methods have been employed. These include thermal treatment, microwave treatment, precipitation, a sol–gel technique, or hydrothermal procedures [83,84]. The attention to natural HAs for bone tissue engineering applications has been generated by their increased biocompatibility, osteoconductivity, and bioactivity [85]. 

### 2.3. Hydroxyapatite Applications

**Figure 4:**
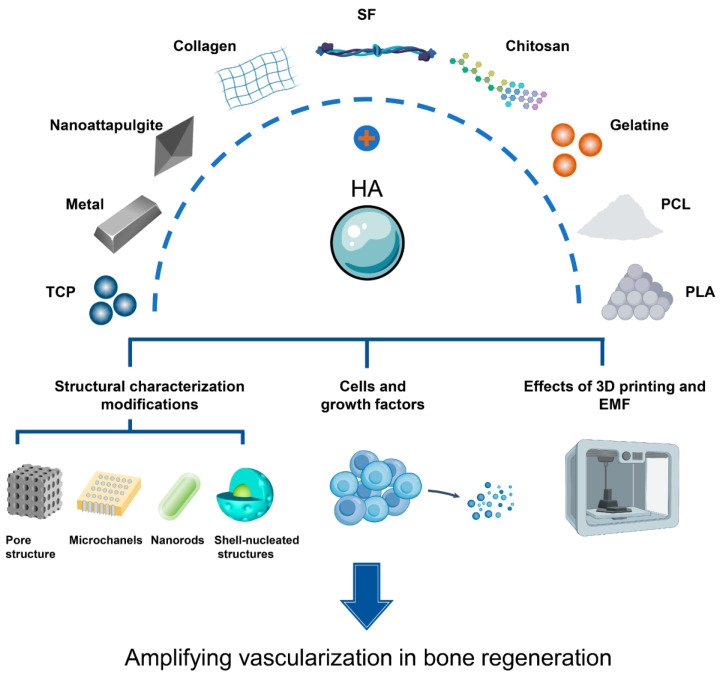
HA incorporation into organic/inorganic materials in order to improve bone tissue regeneration [89].

HA is a promising material for bone tissue engineering that has gained considerable interest in recent years. Its main applications in the biomedical industry have been widely explored due to its unique characteristics such as biocompatibility and enhancing cell proliferation and growth at the implantable site [86]. This material and its composites with polymeric matrices have proved to be ideal, mimicking materials for bone regeneration (Figure 4) due to their intrinsic advantages in osteoinduction, osseointegration, and osteoconduction. Also, HA presents the capacity of undifferentiated cells to render osteocytes and osteoblasts and facilitate the interaction between the osseous tissue and implant. All the characteristics must function properly for the appropriate performance of the application. In this regard, HA can be introduced in a biocompatible and biodegradable polymeric matrix that presents specific architectures and compositions to mimic the natural extracellular matrix. Also, they must possess good vascularization to provide the required transport of metabolic products, nutrients, and bioactive agents [87,88]. 

HA is the most used biomaterial in biomedical applications, offering support for osseous tissue formation and osteoconduction. Many studies have reported its efficiency in maxillofacial applications, orthopedics, infra-bony defects, hip and joint prosthesis, ridge augmentation, as well as in dental applications or coatings. Numerous properties influence their effectiveness, such as composition, size, structure, surface area, shape, and density, which can impact the kinetic process of new apatite formation on the HA surface [90]. HA is also well known for its hydroxyl function group. The groups generate chemical radicals that enhance implant adhesion and implant functionality. Additionally, its improved corrosion resistance has drawn the attention of researchers, as HA-coated metallic biomaterials increase strength and bioactivity. For this reason, numerous researchers have investigated the use of HA in drug delivery, tissue engineering, implants, coatings, and bone regeneration applications [91,92,93]. 

In dental applications, HA is frequently used for tooth fillers to enable bond formation among bone tissue and fillings. Zawisza et al. mentioned that even synthetic apatite, when applied as filling and dental implant coatings, can improve cell adhesion, proliferation, and differentiation, and that bacterial colonization cannot be surpassed [94]. Nano-HA presents several applications in dentistry due to its superior bioactivity and biocompatibility. For example, in cases of dentin hypersensitivity, HA was proven to induce the occlusion of dental tubules by functioning as a mineralizing agent. Likewise, nano-HA is preferred for implant coatings as it enhances bone-to-implant contact and encourages bone adhesion [95]. Another highly studied application involves coating the implant with biomaterials, namely HA, to increase the bioactivity of the implant. Because of its crystallographic composition, chemical structure, and similarity with the native tissue, HA forms a chemical connection between the osseous tissue and the surface of the biomedical implant. This encourages growth stimulation, and, therefore, the osseointegration process [96,97].

Nevertheless, stability in the timing of ceramic coatings affects the success of clinical applications and depends, therefore, on the deposition technique. For example, the deposition method controls the stability and uniformity of the HA bond to the metal substrate. Since CPs are difficult to deposit through direct physical methods, researchers have presented a combination of electrodeposition and 3D printing to avoid direct decomposition. Additive manufacturing techniques have been recently recommended for the incorporation of bioactive agents to adjust their deposition related to the high surface area of the coating material. This method facilitates the deposition of a coating in an efficient manner for drug delivery agents in bone tissue engineering by enhancing the osseointegration and reducing the formation of biofilm on the implant [98,99,100].

Moreover, HA has been also proposed for controlled drug delivery systems, cancer, and gene therapies, as well as bioimaging [101]. Controlled delivery systems can transport drug molecules at a controlled rate and time to increase the efficiency of drug uptake by the human body. Some scientists have proposed the combination of nano-HA with other biomaterials to develop composite bone scaffolds that incorporate drugs. These scaffolds have the capacity to release drugs slowly and locally, thus promoting the formation of new bone in the required zone, but also providing a new opportunity for the treatment of bone diseases. Moreover, they can provide appropriate structures for bone regeneration and support and also shelter the bioactive molecules from environmental degradation factors [102]. Asghar et al. also demonstrated the application of HA in drug delivery systems. They were successful in obtaining HA microporous structures and using them as drug delivery systems. The developed systems can transport bioactive molecules or drugs directly to the specific site [103]. 

Scaffolds containing HA for bone tissue engineering applications can also be considered a successful application in biomedical engineering, acting as an excellent temporary substrate. This substrate allows cell in-growth, proliferation, differentiation, and osseous tissue regeneration after in vivo implantation [104]. Hence, Hou et al. reported that HA with biomimetic micro-/nanostructured hybrid scaffolds possesses the superior adhesion, proliferation, and differentiation of bone marrow stromal cells. Furthermore, the obtained scaffolds had good protein adsorption owing to their surface properties, which contributed to the bioactivity of the composite scaffolds [105].

Lately, HA has been widely used in the fabrication of optical devices, biosensors applications, bioimaging, the coating of metallic implants, and theragnostic applications. At the moment, there are few reports on biosensor applications involving HA acting as an immobilization matrix to allow biomolecule adhesion over an electrochemical surface to provide a suitable microenvironment. Additionally, the excellent biocompatibility and adsorption ability, good sensitivity, and stability of HA favor the material for the fabrication of biosensors [106]. 

There have been numerous applications presented in the biomedical domain which illustrate HA as the ideal material due to its ability to enhance bone regeneration. As HA is known to possess some disadvantages that could lead to the failure of the application, such as mechanical strength or bioactivity, researchers have considered doping the material with different metals to surpass these deficiencies. 

## 3. Comparison of Non-Doped and Doped Hydroxyapatite: Properties, Structure, and Applicability in Bone Tissue Engineering

As mentioned before, HA has been extensively studied due to its extraordinary biological characteristics in the biological environment, being known as the main mineral composition of osseous tissues. HA also presents superior biocompatibility and osteoconductivity, although it is brittle and possesses poor mechanical strength. Therefore, its simple application cannot be used in load-bearing applications even though there are notable differences between natural bone apatite and HA synthetic material. This material provides a hexagonal structure in which the calcium ions are replaced by dopant ions and the structure is distorted. Numerous studies have presented specific results of HA doped with Cu^2+^, Mg^2+^, Zn^2+^, Ag^+^, and many other ions. In this regard, Anushika et al. mentioned that doped HA showed a slight shifting of characteristic peaks. This modification is an effect of the doping agent on the crystal structure. Moreover, the doping ion can lead to distortions in the crystal and lattice parameters. Also, the percentage of crystallite size and crystallinity is reduced [107]. For example, when HA presents substitutions of elemental ions, such as silicate or carbonate ions, the size, crystal lattice, and concentration are modified in order to improve the physicochemical characteristics by altering its surface conditions and electron density. The functions accomplished by the substituted elemental ions have the capacity to modify some mechanisms of the osseous metabolic processes, especially in implantation and bone formation (such as osteoinduction, osteoconduction, and osseointegration). Sugimoto et al. reported that undifferentiated cells were able to mature into osteocytes over time, which is extremely important in implant fixation and bone regeneration [3]. 

The doping of HA can be performed in three different sites in the crystal structure. These include doping at the site of PO_4_^3−^ ions, Ca^2+^ ions, or at the OH^−^ site. Doping with different ions at these sites has been reported to increase the applicability of HA in bone tissue engineering and biomedical applications. Moreover, it has been shown to stimulate bone growth and improve bone density, as well as promote osteocyte proliferation and surpass bone tumor formation [108]. Furthermore, the combined outcome of doping ions is tailored according to the synthesis techniques, synthesis conditions, and doping type used to acquire the desired properties of the materials depending on the required application. The concentration, size, and type of ionic doping agents influence the microstructure, lattice parameters, crystallinity, grain size, crystal morphology, thermal stability, and solubility of HA. There are various types of ions used as doping agents for HA, and numerous studies have proven their influence and efficiency in bone tissue engineering [109,110]. Moreover, Shokri et al. mentioned that the substitution of doping ions in HA structures results in a gradual decrease in the lattice parameters while doping concentration increases. Furthermore, the addition of doping agents limits HA particle growth and reduces crystallinity. On the other hand, the doping of ions also modulates bioactive ion release, which can mediate biological responses [111]. 

Yahia et al. reported, that by comparing normally synthesized HA with doped HA, the crystallite size increases on the doped probe from 15 nm to 62 nm. Moreover, the linear absorption coefficient also shows that doped HA is more absorbable than simple HA, thus increasing its applicability in radiation detection and drug delivery applications. Additionally, the antimicrobial capacity of simple and doped HA on Gram-positive and Gram-negative bacteria and fungi exhibited higher antimicrobial activity of doped HA compared with simple HA [112]. Furthermore, Singh et al. obtained doped HA with Ag, which, in comparison with simple HA, presented comparable hardness, density, and improved antimicrobial characteristics compared to non-doped material. Nevertheless, no difference in cellular functionality, regarding cell adhesion and proliferation, was observed. The doping ion significantly improved the antibacterial property, which is a great feature for biofilm prevention at the implantation sites [113]. Moreover, Aksakal et al. obtained coatings with pure HA and doped with Ag and selenium (Se). The results showed that, while pure HA coatings had an antimicrobial activity of 83% against *E. coli*, the value was increased to 94% with the addition of Ag^+^ ions and 85% with Se ions. Correspondingly, the doping of ions leads to an improvement in HA antimicrobial activity against *S. aureus*. Moreover, cell viability decreased in the simple HA coating but increased significantly in the doped HA coatings [114]. 

Considering antimicrobial activity, pure HA nanoparticles cannot avoid biofilm formation on the surface of the material. This is the main reason for implant and surgical failure. The furthermost known reason for implant failure is a bacterial infection from Gram-negative (*P. aeruginosa*) and Gram-positive bacteria (*S. epidermis*). These infections are difficult to treat and, in most cases, can only be treated by removing the implant, which increases the treatment’s cost and endangers the life of the patient. In this case, doped HA is the ideal solution to enhance the properties of minerals introduced into the biological environment and enhance clinical outcomes. This route offers the opportunity to mimic the conditions of biological apatite. The biological apatite is generally found carbonated and substituted with trace amounts of ions. Consequently, some HA dopants can substitute similar roles as growth factors. Due to these improved properties, doped HA nanoparticles are considered in biomedical applications as antimicrobial agents, bioimaging probes, drug carriers, and theragnostic agents [115]. In this regard, Jian et al. reported that the use of HA in drug delivery systems is highly encouraged due to its excellent adsorption capacity and biocompatibility. They demonstrated that, compared with pure HA, doping ions not only improved its zeta potential and specific surface area but also enhanced the binding energy between the bioactive compound and HA, thus increasing its drug-loading capacity and prolonged release rate [116].

Considering their mechanical properties, the incorporation of the doping agent may lead to an increase in strength and corrosion resistance. In this direction, Vladescu et al. proved that substitution with doping ions improves the hardness and elastic modulus [117]. Moreover, Balakrishnan et al. mentioned that, by comparing pure HA and doped HA, the durability of the material has increased. Furthermore, by enhancing the concentration of the doping agent, the results of a Vickers microhardness test demonstrated increased hardness [118]. Additionally, Manzoor et al. confirmed that, by increasing the doping content, Young’s modulus of the obtained material increased [119]. The concentration of the doping ions has a significant influence on both the mechanical properties and the type of ions. For example, Aryal et al. demonstrated that Mg^2+^ substitution into an HA structure enhances the elastic properties of the material; meanwhile, Zn^2+^ degrades it [120]. In another study, a research group led by Wang studied the influence of doping ions (F^−^) on an HA structure. They established that F^−^ both restores the mechanical properties of the HA lattice and successfully prevents the dissociation of the hydroxyl groups on its surface. Even though the doping ions cannot directly stop the calcium loss, they can indirectly reduce this process [121].

This substitution can be performed either with only one type of doping ion or with a co-substitution of two or three doping agents. In this case, there can be a combination of different doping material properties to improve the functionality of the biomaterial applied in bone tissue engineering. The co-substitution of the doping ions in the HA lattice may lead to the optimization of osteoconductivity, thus facilitating novel osseous tissue formation and increasing osteoinductivity, with the promotion of the differentiation of the mesenchymal stem cell to the osteoblast. These improved biological effects have been reported to inhibit osteoporosis. Ullah et al. reported that both co-doped ions also improve the solubility of the developed material and present an antimicrobial response, depending on the doping ions, as well as prevent bacteria growth [122,123]. As an example of co-substitution effects, Cao et al. developed three different coatings: the Mg-doped HA coating, F-doped HA coating, and HA doped with both F^−^ and Mg^2+^ coatings. After thorough investigations, the in vitro bioactivity studies presented different microstructures on the three coatings after soaking them for 7 days: a rice-like, nano-porous structure on the coating doped HA with F^−^, a 3D porous network structure on the coating doped with Mg^2+^, and an ordered linear structure similar to the cancellous bone for the coating doped with both elements. The coating doped with both elements presented improved transformation from apatite-like to HA phase because of the synergistic effect of F^−^ and Mg^2+^. Moreover, cell viability analyses indicated that co-doped HA coating possesses good biocompatibility with no cytotoxicity and improved interfacial bioactivity in the titanium substrate, as well as good cellular proliferation [124]. 

## 4. Doped Hydroxyapatite: Doping Material and Methods, Synthesis Method, and Properties

**Figure 5:**
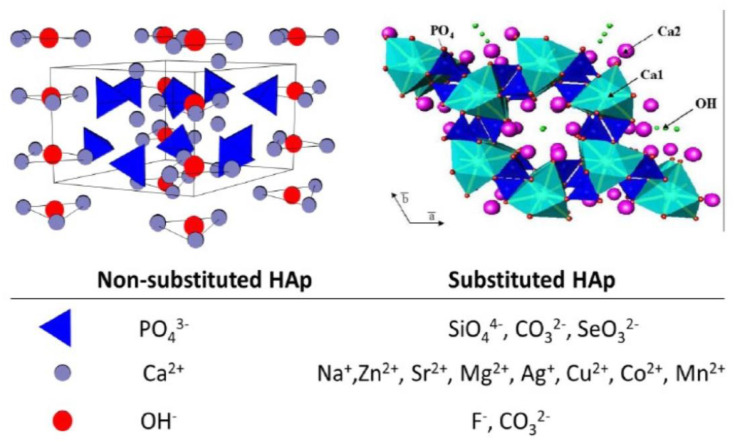
Representation of doped and non-doped HA. Reproduced with permission from the Royal Society of Chemistry, 2012 [38].

HA is well known for its applications in the biomedical field due to its similarity with native tissue. Even though it possesses good characteristics for its use in bone tissue engineering, this material limits its use in load-bearing applications due to its poor mechanical strength and reduced bacterial resistance. To surpass these deficiencies, in the structure of HA, metal ions can be incorporated, such as Y^3+^, Zn^2+^, Mn^2+^, Mg^2+^, or Ag^2+^ (Figure 5). They are substituted in low concentrations into the HA lattice to improve biological capacities [125,126,127]. An ideal dopant must enhance the physicochemical characteristics of HA without negatively influencing the biological performance of the doped material. Additionally, small quantities of dopant agents must provide a superior combination of strength and elastic modulus to significantly improve HA fracture toughness [20].

Metal ions and their oxides have gained increased attention due to their superior antibacterial properties, especially against bacterial resistance to antibiotics. In contrast with antibiotics, metal ions function differently in bacterial and mammalian cells because of the deviation of metal transport systems from usual patterns. This enables the use of doped material with ions as antimicrobial agents with no negative influence on the human body [128]. These metal ions have three main mechanisms for antimicrobial properties. Firstly, metal ions will bind to proteins and then deactivate them. Secondly, metal ions will further interact with the microbial membrane, which provokes structural modifications and permeability. Finally, metal ions will interact with the microbial nucleic acids and then prevent microbial replication [129]. 

Furthermore, by doping the material with other materials, modifications in the lattice parameters, crystallinity, thermal stability, and morphology are performed, significantly influencing the solubility of the doped HA in contact with the physiological environment. Moreover, the structure of the developed biomaterial can offer efficient support for the improvement of bone tissue growth and angiogenesis on the implant [130,131,132]. Thus, methods must be further developed to adjust the structure of stoichiometric HA to enhance the bioactivity of the biomaterial [133]. 

The substitution with other ions in the structure of HA has been shown to enhance osteoblast differentiation, osteoinductivity, osteoconductivity, and osseointegration, as well as the ability to release the substituted ions because of the reduced solubility in HA (Table 2). The doping of Cu^2+^, SiO_4_^4−^, and Mg^2+^ has demonstrated the improvement of osteoinduction, while the introduction of Na^+^, Cl^−^, or CO_3_^2−^ can enhance osteoconduction. Additionally, HA doped with Sr^2+^ or Zn^2+^ improved several biological properties, such as the suppression of bone resorption and osteoporosis and the enhancement of bone formation. Moreover, researchers have reported that HA doped with SiO_4_^4−^ enhances the dissolution rate and osseointegration properties. On the other side, F^−^-doped HA has been used to treat osteoporosis; however, high concentrations of F^−^ can lead to osteosclerosis and other diseases [127,134].

### 4.1. Zn^2+^-Doped Hydroxyapatite: Doping Method, Properties, and Biologic Activity 

Zinc (Zn) is present in osseous tissues as a trace element, which has a great connection with bone metabolism. Furthermore, Zn^2+^ possesses antioxidant properties and is extremely important in various biochemical processes, such as blood clotting, protein digestion, or carbohydrate metabolism. Moreover, it has a substantial impact on the immune system. In this regard, numerous studies have indicated that Zn could promote osteoblasts cell proliferation and differentiation, as well as enhance bone formation. Considering these properties, its incorporation into HA may improve the bioactivity in bone tissue engineering [135,136]. Zn substitution in HA structures has gained great interest due to its presence in bone and teeth enamel, influencing the metabolic processes for bone growth. The incorporation of Zn ions improved bone development, biomineralization, and osteoblast production. Nevertheless, the lack of Zn in the osseous tissues reduced bone density [137]. 

The latest reports show that the presence of Zn in ceramic materials provides antimicrobial activity, depending on the concentrations of the doping material. Buyuksungur et al. reported that a small concentration of Zn in the structure of HA is sufficient to ensure resistance against *S. aureus*, *K. pneumoniae*, or *B. cereus* [138]. Comparable results were also mentioned by Ofudje et al., who Zn-doped HA, which was prepared using the chemical co-precipitation method and further used as a scaffold. Their bioactivity and antibacterial activity against *E. coli* and *S. aureus* were successfully demonstrated. Moreover, researchers have highlighted that, by increasing the Zn concentration, the dissolution rate increases due to a decrease in crystallinity [139]. Candidato Jr. et al. also mentioned that Zn incorporation does not alter the morphology of coatings, while X-Ray Diffraction analysis showed that a Zn concentration higher than 10 mol% can inhibit HA formation and encourage calcium zinc phosphate formation. Moreover, they confirmed that Zn ion substitution affected the crystal structure and changed the crystallinity and lattice parameters due to lattice distortion [140]. 

Further, Zhou et al. proved that the Zn-doped HA, when applied as a coating on the surface of the implant, not only yielded higher corrosion resistance but also enhanced bacterial inhibition and osteogenic differentiation. Compared to pure HA coatings, doped coating not only successfully inhibited bacteria, but also promoted cellular adhesion and differentiation of bone marrow mesenchymal stem cells [141]. Moreover, Sergi et al. demonstrated that Zn-doped HA prepared by co-precipitation nibbled the modulation of osteoblast activity and antibacterial capacity. The developed materials have been applied as coatings, and their bioactivity was proved by soaking them in SBF. After further investigations, the coatings exhibited non-cytotoxicity against human osteoblast Saos-2-cells and antibacterial effects against *S. aureus* and *E. coli*, with higher efficiency against the Gram-positive bacteria [142]. In another study, Popa et al. investigated the application of Zn-doped HA on the collagen matrix. After biological evaluations, the developed structures showed no toxicity in bone tissue engineering applications. Moreover, doped HA can be effectively applied in drug delivery systems. In this regard, Venkatasubbu et al. reported high efficacy in the control of the drug delivery rate of Zn-doped HA that incorporated ciprofloxacin as a bioactive molecule [143].

### 4.2. Mg^2+^-Doped Hydroxyapatite: Doping Method, Properties, and Biologic Activity 

Magnesium (Mg) is another preferred substitute for HA due to its chemical similarity. Mg ions are acknowledged for bone mineralization promotion and the enhancement of osteoblast-like cell proliferation; hence, they are also known for enhancing the biocompatibility and bioactivity of a developed material [144]. Further, Mg is the fourth most highly concentrated cation which can be found in the human body after Ca, P, and Na. The deficiency of Mg in osseous tissue has been suggested as a possible risk factor for the generation of osteoporosis. In the human body, Mg^2+^ has the capacity to reduce the degradation rate of CPs and influences the crystallization of mineral substances. In this regard, the introduction of Mg ions into HA encouraged endothelial cell and osteoblast resistance, spreading and enhancing osteoblast adhesion and cell proliferation [133]. 

The substitution with Mg^2+^ ions not only led to the modification of biological properties but also of the mechanical and chemical structure. Pana et al. mentioned that the addition of doping ions into the HA matrix improves the hardness and elastic modulus compared to the pure HA [145]. Further, Monte et al. mentioned that Mg^2+^ also affected the crystal structure, reduced its porosity, and enhanced the bending strength and corrosion resistance [146]. To confirm this, Veljovic et al. developed bone fillers from Mg-doped HA, highlighting the interaction between the human osteoblast, mesenchymal stem cells, and implant material, as well as the in vitro and in vivo formation of the novel osseous tissue [144]. Moreover, Lv et al. investigated the cytotoxicity of doped HA to osteosarcoma cells (MG-63) and mesenchymal stem cells (MSCs). After biological investigations, HA doped with Mg ions was found to enhance the adhesion and proliferation of MG-63 and MSC cells, promoting their metabolism. A suitable concentration of the doping agent improves material biocompatibility [147].

Daood et al. also developed a dentine adhesive modified with Mg-doped HA. The doped material was also prepared using precipitation methods. After thorough investigations, it was proven that the doped biomaterial possesses antibiofilm and self-remineralization properties [148]. Moreover, Jenifer et al. also reported an improvement in antibacterial and hemolytic efficiency after the substitution of Mg ions into the HA structure. The incorporation of doping ions also proved to reduce inflammatory activity [149].

Zhao et al. tried a new method for obtaining Mg-doped HA with oriented microchannels through hot-pressing sintering to provide uniformity and an arranged control of pores. Compared with normal synthesized HA, the doped material exhibited superior mechanical and biological properties. The density and microchannel size extraordinarily influenced the compressive strength of the doped bioceramic. Additionally, the microchannel-containing ceramic exhibited improved apatite mineralization in SBF [150].

### 4.3. Cu^2+^-Doped Hydroxyapatite: Doping Method, Properties, and Biologic Activity 

Lately, copper (Cu) has been recognized to exhibit good biological properties by enhancing the differentiation of mesenchymal stem cells into osteoblast cells, facilitating endothelial cell proliferation in wound healing, reducing implantation infections, and increasing the gene delivery levels of vascular endothelial growth factors. Considering all these aspects, Cu^2+^ is an ideal element to exhibit specific characteristics for bone repair; however, at the same time, the physicochemical characteristics of Cu-based materials are altered. Moreover, excessive Cu release into the human body may cause cytotoxicity [151]. Furthermore, Hidalgo-Robatto et al. demonstrated that doped HA promotes osseointegration at the interface between the bone and implant surface and simultaneously inhibits bacterial biofilm formation. Finally, the doping of HA with Cu^2+^ has numerous advantages to be further applied in metallic implants for the regeneration or repair of severely damaged or fractured bone tissue. Moreover, not only was biocompatibility increased, but the mechanical stability of the doped HA was also increased, which is a desired improvement for numerous biomedical applications [152]. On the other side, Ercan et al. mentioned that the addition of Cu altered the thermal stability, morphology, and agglomeration degree [153]. 

**Figure 6:**
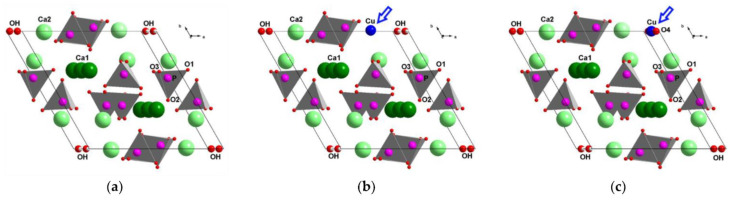
Schematic representation of stoichiometric HA structure (**a**) of Cu–HA (blue arrow), featuring the localization of the copper cation, either at the site of the calcium cation (**b**) or in the hydroxyl channel. (**c**) The arrow indicates the position of the copper atom [156].

Cu^2+^ ion substitution into the HA structure (Figure 6) presents a beneficial effect not only on angiogenesis, cell proliferation, and the differentiation of stem cells but also on improved cell migration while inhibiting osteoclast activity [154]. To further investigate the biological activity, Ressler et al. obtained Cu-doped HA using the precipitation method, with the substitution of Cu^2+^ at Ca sites. They also tried the immersion method of non-substituted HA in a solution of Cu^2+^ ions, with the substitution occurring at OH sites. While high concentrations of Cu^2+^ presented good antibacterial properties, toxicity was also reported; therefore, a suitable concentration must be selected in order to also illustrate cell proliferation [155].

Furthermore, researchers have reported that Cu incorporation into ceramic materials leads to the improvement of neovascularization and stimulation of endothelial cell proliferation in vitro [157]. Park et al. performed in vitro studies which confirmed the nontoxic effect of both doped and non-doped HA using MG-63 cell lines. The developed materials successfully simulated and facilitated new cell attachment, growth, and proliferation on the surface of the implant with adequate compressive strength [158]. In another study, I’Alzit et al. incorporated Cu ions into HA using thermal treatment. The obtained materials exhibited the capacity to stimulate blood vessel formation, together with the enhancement of mechanical properties and antibacterial properties [159]. 

### 4.4. Sr^2+^-Doped Hydroxyapatite: Doping Method, Properties, and Biologic Activity 

Strontium (Sr) is another trace element that is present in the osseous tissue. Sr presents outstanding bone-healing properties and offers therapeutic significance for the treatment of osteoporosis. Sr^2+^ ions present a dual importance in stimulating bone regeneration, involving the inhibition of osteoclast activity and the stimulation of osteoblasts, leading to new bone formation and a decrease in bone resorption. It was proved that Sr has a high influence on bone density and improves bioactivity and mechanical properties [160]. 

Many studies have used Sr-doped HA coatings on the surface of the implant to increase the bioactivity and reduce the corrosion in time. This trace element significantly decreases the risk of bone fractures and the generation of osteoporosis. Further, Sr inhibits bone resorption by reducing the adsorption activity of osteoclasts and differentiation; moreover, at the same time, it reduces the production of osteoblast matrix metalloproteinase [160,161]. Furthermore, the introduction of dopants in the HA structure influences microstructure, morphology, solubility, thermal stability, and the mechanical properties of the bioceramic; therefore, it is a good method to obtain specific properties for the biomaterial in conformity with the desired application [123]. As mentioned before, the incorporation of Sr ions into the structure of HA modifies not only bioactivity but also physicochemical properties. In this regard, Baldassarre et al. obtained Sr-substituted HA powders with different Sr concentrations that were prepared using a solid-state synthesis method. After thorough investigations, researchers proved that, by increasing the concentration of the doping agent, the particle size and distribution were modified. Further, their substitution led to lattice expansion due to the larger Sr atoms. Moreover, by increasing the Sr concentration into HA, PO_4_^3−^ groups were broadened due to the enhancement of the crystal structure disorder and the decrease in the crystallite size [162].

In the last few years, researchers have focused on the development of Sr-substituted HA. The doped materials were proved to enhance the bioactivity of the material as Sr is known to stimulate bone formation. Generally, Sr-doped HA is a hydrophilic and alkaline material with superior solubility and good compressive strength, which is similar to native tissue mechanical properties [133]. Hu et al. mentioned that a low concentration of Sr^2+^ presents great efficiency in stimulating osteoblast proliferation and differentiation, while a higher concentration negatively impacts the bone mineralization process. Thus, the selection of a suitable concentration for the doping agent is essential for bone tissue engineering applications [163]. Moreover, Wang et al. mentioned that Sr^2+^ substitution also influences the morphology and size of the obtained material. The immersion and electrochemical tests also showed that Sr doping improved the corrosion resistance of HA coatings. Moreover, BMSCs’ cell culture results highlighted that Sr^2+^ addition promotes cell adhesion and the proliferation of HA-coated Mg alloys [164]. In another study, Xu et al. obtained Sr-doped HA microspheres with interconnected porosity and pore channels, prepared via the hydrothermal method. They reported that the morphologies, constituents, scales, and pore geometries of the obtained materials were modulated by the concentration of Sr^2+^. The substitution of Sr^2+^ significantly enhanced the biodegradability of the microspheres. Additionally, the porous microspheres presented a superior drug loading capacity and sustained release properties. These particularities have also proven the potential of Sr-doped HA to be applied in drug delivery systems [165].

### 4.5. Ag^+^-Doped Hydroxyapatite: Doping Method, Properties, and Biologic Activity 

Silver (Ag) is another well-known element to be incorporated in numerous materials due to its pronounced antibacterial activity, biocompatibility, and low toxicity at reduced concentrations [166,167]. Ag^+^ was selected in numerous applications in the biomedical field for its increased bioactivity and thermal stability. Further, Ag^+^ presents the ability to attach to proteins and enzymes in bacterial cells and inhibit the main cell functions of various Gram-negative and Gram-positive bacteria, including *S. aureus* and *E. coli*. Ag ions incorporated into the HA structure would generate a multi-functional adsorbent material [168].

Antibacterial activity is the primordial characteristic of Ag^+^ to be considered for its use as a doping agent, as it possesses the capacity to control biofilm formation and bacteria adhesion. Many studies have demonstrated the efficiency of the use of Ag-substituted HA in bone tissue engineering. The doped materials can be obtained using hydrothermal, precipitation, and mechanochemical techniques [169]. The incorporation of Ag ions into the HA structure to develop materials with tailored properties that meet the functionality and quality requirements for a bone substitute has been the main goal. These doping ions have been preferred due to their superior antimicrobial activity against fungi, viruses, and a wide spectrum of bacteria. However, their applicability is strongly influenced by the toxicity of metal ions. Taking this into consideration, the biocompatibility of doped HA can be partially affected. Nenen et al. mentioned that, compared to other metallic ions, the use of Ag^+^ ions as a doping material in biominerals can still show lower cellular stress [170,171]. In this direction, it has been reported that positively charged Ag nanoparticles exhibited higher antimicrobial activity than negatively surface-charged nanoparticles. Yet, the cytotoxic effect of these nanomaterials may relate to more than one physicochemical characteristic. Undeniably, even though many researchers focused their interest on the antibacterial property of Ag nanoparticles, the mechanisms involving the bactericidal property are still under continuous research [172].

Chatterjee et al. successfully developed Ag-doped HA, which was prepared using an in situ hydrothermal method. The obtained material confirmed the effective doping of Ag ions in the HA lattice, which influenced the thermal stability of the material and ensured significantly high ionic conductivity with reduced activation energy for increased temperatures. Moreover, the superior biocompatibility of the Ag-doped HA rods and good electric and dielectric properties highlighted the capability of the obtained material as a nanocarrier for electric field-induced drug delivery systems in the future [173]. Moreover, Citradewi et al. mentioned that, by doping the material with Ag ions and using a natural source for the synthesis of HA, the bioactivity of the material could be further improved compared to the simple doped HA. Moreover, the bacteriostatic properties were enhanced over time [174]. Regarding physicochemical properties, Zhou et al. demonstrated that Ag could be absorbed on the surface of HA. This led to morphological modifications, excluding the enhancement of biocompatibility and antibacterial activity [175]. 

### 4.6. Hydroxyapatite Doped with Other Metals: Doping Method, Properties, and Biologic Activity

There are many other metals and composites that have been studied as doping materials to be incorporated into the structure of HA. Depending on the desired applications and required properties, they have been extensively investigated to improve the mechanical properties and bioactivity of the designed material to be used in the biomedical domain.

Iron (Fe^3+^) is another doping agent that is present in trace quantities, and it is known for its importance in bone health. When Fe is incorporated into the structure of HA, the physical and biological properties are improved. Fe-doped HA is known to be synthesized through different routes like spray drying and hydrothermal and ultrasonic irradiation [176]. 

Another doping material highly studied in biomedical applications is represented by cerium (Ce). Ce^3+^ ions possess good antimicrobial abilities and low toxicity. This material is highly recommended for its bioactivity, such as its capacity to improve the reproduction of collagen, as well as the proliferation, differentiation, and mineralization of osteoblast cells [177]. Sousa et al. mentioned that Ce^3+^ ions can be easily incorporated into HA structures due to their capacity to occupy Ca^2+^ sites, as the Ce^3+^ ionic radius is similar to that of Ca^2+^. This substitution demonstrated the enhancement in bioactivity, especially regarding cell viability, antibacterial capacity, an increase in metabolic rate, and antioxidant activity [178]. Moreover, Padmanabhan et al. mentioned that Ce doped-HA encouraged the deposition of calcium-rich apatite layers on the surface of SBF, mimicking the hardness of natural bones [179]. Additionally, Nisar et al. demonstrated that Ce substitution into the HA structure also improved mechanical properties [6]. 

Chromium (Cr) is another trace element in the human body that is essential to metabolize fats and sugars. Cr^3+^ doping is also used to enhance the hemocompatibility of HA. Hence, Cr doping is very advantageous for biomedical applications; however, the cytotoxicity needs to be properly assessed [180,181]. To confirm the applicability of Cr in bone tissue engineering, Lima et al. obtained Cr-doped HA powders with different Cr^3+^ concentrations through the co-precipitation method. After thorough investigations, the results showed that the modifications of the mechanical properties are influenced by the doping concentration [182]. Iqbal et al. also confirmed that, by increasing the Cr ion concentration, the crystallinity of the sample decreases [183].

Cobalt (Co) is an indispensable trace element with a very specific function in the human body. Yet, some studies involving Co-doped bioceramics have demonstrated the angiogenic effect of Co^2+^, leading to the enhancement of the neovascularization of new bone formation [184]. Kulanthaivel et al. also obtained Co-doped HA that was prepared using the precipitation method with different doping concentrations. Researchers proved that mechanical properties are altered depending on the concentration of the doping ions. Further, they demonstrated that Co doping into an HA structure presented great proangiogenic properties without harming the osteogenic property of the HA [185]. 

In another study, Abutalib et al. investigated the application of Molybdenum (Mo) as doping material. They successfully obtained doped and non-doped HA using the efficient microwave technique at low temperatures. After biological investigations, they demonstrated and improved antibacterial activity with the addition of the doping agent, confirming their applicability in biomedical applications [186].

## 5. Latest Applications of Doped Hydroxyapatite in Bone Tissue Engineering

HA has attracted immense attention in biomedical applications due to its similarity with natural osseous tissue, high bioactivity, osteoconductivity, and biocompatibility. Even though this material has great advantages, there are still some drawbacks that need to be solved for it to properly function in bone tissue engineering. As the mechanical properties and antibacterial ability still need to be improved, HA doping has been the ideal solution to solve all issues, depending on its applicability. 

### 5.1. Applicability of Doped Hydroxyapatite in Dental Applications

Many studies have focused on the development of ion-doped HA for the treatment of numerous dental applications. In this direction, many researchers have focused on the use of HA, which is the main tooth constituent that exhibits a remineralizing effect on tooth enamel; moreover, it can enhance the bond’s strength to the structure. As HA still lacks mechanical strength and bioactivity, the application of doped HA was the ideal solution for solving some drawbacks that may appear during the treatment time. Thus, doping aids the obtained materials with enhanced antibacterial properties to stimulate new bone growth and prevent inflammation, which is highly important in dental applications [187]. Tautkus et al. mentioned that orthodontic materials containing doped ceramics (such as Zn^2+^ ions) demonstrated higher antibacterial and remineralization effects compared with non-doped materials. Further, these doped materials are innovative biomaterials for filling bone cavities as a matrix for cells to support the regeneration of damaged tissue [188].

Mathew et al. obtained Sr-doped HA remineralizing paste, with and without chitosan, in order to investigate the antimicrobial capacity. The antibacterial efficacy of the developed paste was improved by the addition of chitosan, and its antimicrobial efficacy was comparable to calcium hydroxide. Moreover, the antibacterial efficacy of Sr^2+^-doped HA paste with and without chitosan was assessed against the most common microorganism involved with the process of dental caries (*S. mutans*). As Sr^2+^ is a trace element in the human body, it can be easily incorporated and accepted by tissues. The doped material presented different doping ion concentrations. After performing the investigations, researchers showed the ability of Sr-doped HA to induce an improved formation of biomimetic apatite both in SBF and cell culture medium. Moreover, the incorporation of Sr into the HA structure increased the antibacterial capacity of the material [189]. 

In another study, Sauro et al. investigated the chemical, structural, and biological effects of fluoride-doped HA (F-doped HA) with different doping ion concentrations as potential remineralizing materials for dental applications. Thus, researchers have mentioned that mechanical properties have been modified and that all obtained materials were able to produce F-containing, apatite-like crystals after SBF immersion. Also, by increasing the concentration of F^−^ ions, there will be a prolonged release of F^−^ ions and increased precipitation of CaF_2_; thus, there will be high biocompatibility [190]. Teerakanok et al. also explored the influence of different doping ions (Mg^2+^, F^−^, and Zn^2+^) on the structure of HA by investigating crystal organization, crystalline phases, and morphology in enamel remineralization. The researchers mentioned that, by increasing F^−^ concentration, the shape and size of HA crystals were affected; meanwhile, Mg^2+^ did not directly affect the morphology of HA crystals. Moreover, in the presence of Mg^2+^ combined with F^−^, small needle-like crystals with a higher density were formed. Also, considering the incorporation of Zn^2+^ ions, they mentioned that increased concentrations led to the inhibition of crystal formation. Furthermore, by combining F^−^ with Zn^2+^ in low concentrations, dense needle-like crystals formed homogenous layers. As a result, they have proven the synergistic effect of combining different doping ions into the crystallization of HA structures in enamel remineralization [191]. 

Moreover, Ciobanu et al. investigated the applicability of Ce-doped materials as coatings on dental implants. The doped coatings presented good antibacterial efficiency against *S. aureus* and *E. coli*, with higher efficiency against *E. coli*. [192]. Additionally, Kadhim et al. studied other doping ions, such as Sr^2+^ and Mg^2+^, to obtain HA coatings to be deposited on Ti–6Al–4V dental alloys. They proved that both doping materials improved mechanical and biological properties. Moreover, the corrosion resistance of the dental alloys in the saliva environment was also improved [193]. 

### 5.2. Applicability of Doped Hydroxyapatite in Drug Delivery Systems

Considering dental or orthopedic interventions, HA is the optimum material used for drug delivery systems to directly transport the active substance in the affected zone. This may lead to a reduction in the treatment time with the antibiotics to avoid an overdose. In this regard, HA has been preferred as a drug carrier due to its controlled release rate. The crystallinity, porosity, phase purity, particle size, morphology, solubility, agglomeration tendency, and chemical composition of HA have also influenced the success of the drug release at a prolonged rate [194]. However, HA still has some inherent limitations, considering its mechanical and antibacterial properties. In this direction, the biological, physio-chemical, and antibacterial characteristics of synthetic HA can be tailored with the aid of doping/substitution with several selected ions. The exceptional HA structure offers extensive stability and flexibility for the substitution of various ions into its lattice. Many examples in the literature have demonstrated the efficiency of doping the materials and their further use in drug delivery applications. Numerous anions (SiO_4_^4−^, CO_3_^2−^, Cl^−^, F^−^, HPO_4_^2−^) and cations (Mg^2+^, Sr^2+^, Zn^2+^, Mn^2+^, Na^+^, Ce^3+^, K^+^, etc.) have been investigated for doping to obtain materials with desired properties [195].

Therefore, Ullah et al. prepared Sr^2+^/Fe^3+^ co-doped HA to be further applied in drug delivery systems. After their synthesis, an increase in lattice parameters was detected with a high degree of Sr^2+^ substitution and a reduced degree of Fe^3+^ substitution. This variation in lattice parameters demonstrated that both incorporated ions partially substitute Ca^2+^ ions in the HA lattice. The incorporation of both Sr^2+^ and Fe^3+^ improved the biomechanical properties; therefore, it has been proposed as a suitable candidate for drug delivery systems and as a heat mediator for hyperthermia [155,196]. De Lama et al. proposed the direct incorporation of SeO_4_^2−^ ions in an HA structure for the prevention of bone cancer metastasis and for the control of skeletal tumors. They suggested that the cytosolic release of Se activates a caspase-dependent apoptosis pathway that is complemented by the formation of reactive oxygen species. Furthermore, the combination of Se with Pt-based drugs led to a reduction in nephrotoxicity and bone marrow suppression, effects which are attributed to the formation of intermediate Se–Pt complexes [197].

Furthermore, Febrian et al. demonstrated the efficiency of doping agents in drug delivery systems applied for cancer treatment. The substitution of HA with specific doping ions (such as Zr^4+^) improved the efficacy of HA in cancer treatment. Doped HA was assessed for cellular uptake, cytotoxicity, and biodistribution pattern evaluation. They demonstrated that, by doping HA, the efficacy of HA in lung cancer therapy was improved [198]. In another study, Jose et al. developed doped Mg to be further applied in drug delivery systems. The substitution has been demonstrated by the changes in the lattice parameters of HA. Moreover, the results showed that, by increasing the concentration of Mg, the morphology of the samples was altered with smaller particle sizes. Further, amoxicillin was loaded, and its release studies were performed on pure and Mg-doped HA using in vitro conditions. Moreover, researchers demonstrated that, by doping HA, the mechanical properties and bioactivity were improved enough to further use the material in drug delivery systems [199].

Zheng et al. obtained Sr-doped HA microspheres using a facile solid-state reaction and subsequent hydrothermal treatment to prepare mesoporous structures. The prepared microspheres possessed a superior drug-carrying ability and prolonged release performance. Moreover, the microsphere’s in vitro cytocompatibility was evaluated, revealing the good cytocompatibility of the microspheres. Moreover, the prepared Sr–HA microspheres offered an effective solution with which to deliver a stable source of drugs and therapeutic ions to promote bone repair and regeneration [200].

### 5.3. Applicability of Doped Hydroxyapatite in Bone Tissue Engineering

HA is one of the most frequently used bioceramics in bone tissue engineering due to its good bioactivity and superior biocompatibility. Yet, HA presents low fracture toughness, weak wear resistance, low solubility, and difficulty in processing steps into three-dimensional shapes, which limits the variety of applications. Numerous researchers have prepared and designed composites by combining HA with other materials such as bioactive glass, polymers, and carbon materials to surpass the above problems. Moreover, to increase the bioactivity, mainly the osteogenic properties, doping ions were incorporated into the structure of HA [201].

To prove the abovementioned aspects, Ai et al. obtained a Cu-doped HA sodium alginate composite scaffold that was developed using 3D printing. They demonstrated a uniform distribution of Cu^2+^ into the scaffold. Thus, after mechanical behavior studies, they observed that doped Cu ions improved the compressive strength of the scaffolds compared to the undoped Cu scaffolds. Combined with the shrinking results, the incorporation of Cu^2+^ improved the densification of the obtained scaffolds, resulting in the enhanced compressive strength of the scaffolds. Regarding biological activity, the developed scaffolds demonstrated low cytotoxicity and good antibacterial properties, resulting in their recommendation for the developed material in bone tissue engineering [202].

In another study, Banerjee et al. used erbium ion-doped HA to obtain luminescent chitosan–HA nanocomposite films to be applied as antimicrobial dressing materials, implant materials, or fluorescent cell regeneration scaffolds. The developed scaffolds presented good flexibility, facile processing, excellent antimicrobial activity and bioactivity, and fluorescent properties under in vitro conditions [203]. Yuan et al. also obtained composite scaffolds from chitosan and Sr-doped HA with different concentrations. They were further applied as coatings, with good density and uniformity, ductility, and improved adhesion. Sr incorporation led to crystal growth and increased grain size. Moreover, an increase in the concentration of the doping ions improved biocompatibility, enhancing osteoconductivity, cell adhesion, and proliferation [204]. 

Furthermore, Monte et al. developed scaffolds based on alginate and Mg-doped HA, considering that the incorporation of Mg^2+^ ions and alginate in HA leads to an improvement in bioactivity. The composites of the obtained materials presented great potential for bone tissue regeneration. The (Ca + Mg)/P decreased ratio (<1.67) showed that the inorganic phases of the composites were more soluble in the physiological medium compared to HA; therefore, the scaffold had higher bioactivity and bioavailability with the incorporation of Mg^2+^ [146].

Many studies have attested to the influence of doping ions on HA structures by improving the bioactivity of the developed material. Likewise, the doping process has successfully led to enhanced bone formation and regeneration with no cytotoxic effects, characteristics that hugely impact the prevention of infections, or other diseases that may derive from orthopedic implant surgeries.

## 6. Future Challenges, Perspectives

HA synthesis and its application in biomedical engineering have attracted significant interest in the development of an ideal material that presents mechanical strength, superior biocompatibility, and bioactivity. Although pure HA lacks sufficient mechanical strength to be applied in load-bearing applications and has reduced antimicrobial activity, researchers have focused on finding solutions to surpass these limitations. Ion doping/substitution is the perfect solution to introduce trace elements in HA structures and modify the properties of the material without compromising the biological performance of the doping ion. Influenced properties include morphology, size, crystallinity, mechanical strength, particle distribution, and especially bioactivity. In this regard, the optimum concentration and type of doping material are still in need of further research, as a high concentration can disturb bioactivity or even induce toxicity. 

**Figure 7:**
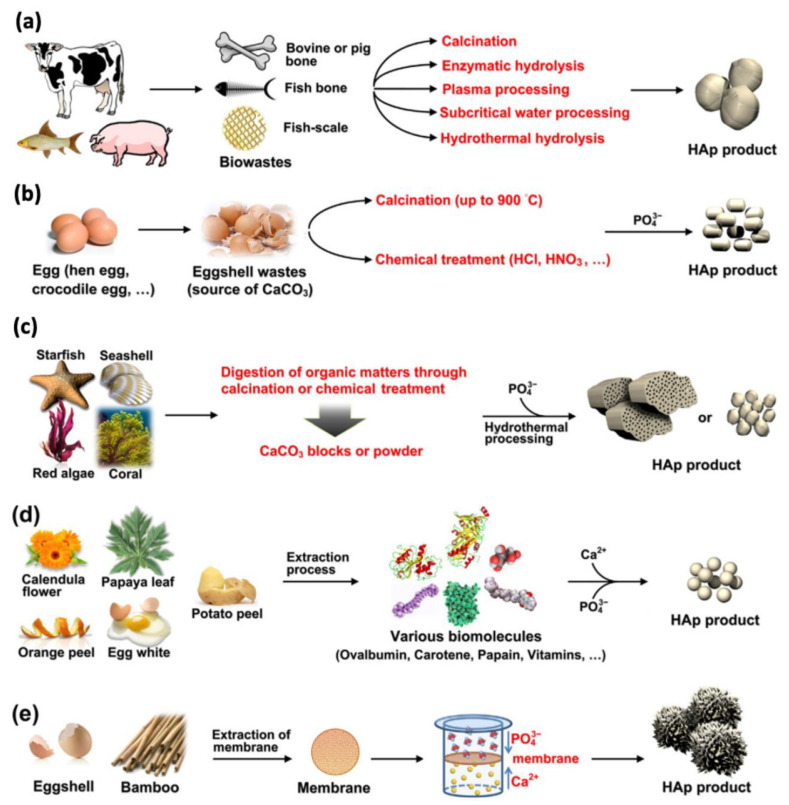
Natural sources used to obtain HA: (**a**) biowaste/animal bones; (**b**) eggshells; (**c**) marine organisms; (**d**) naturally derived biomolecules; (**e**) biomembranes. Reproduced with permission from Elsevier, 2013 [206].

Moreover, there is still a necessity for further investigation to correlate the synthesis methods of HA, its physicochemical properties, and bioactivity to be used in bone tissue engineering. Moreover, the effects of the doping agents have not been fully demonstrated, as most researchers have focused only on the development of the synthesis methods. This also offers the possibility to consider future research on the functionalization of the obtained HA with more than one dopant to study the synergy between doping materials [35]. Lately, researchers have focused on the synthesis of HA from natural sources (Figure 7), as they are known to contain trace elements such as Na^+^, F^−^, Zn^2+^, K^+^, Mg^2+^, Si^2+^, Ba^2+^, and CO_3_^2−^ that mimic the apatite from the human bone. The application of HA extracted from natural sources is considered to be a sustainable, environmentally friendly, and economical process [61]. The HA obtained from biogenic sources permits the substitution of trace elements at the PO_4_^3−^, Ca^2+^, and OH^−^ sites of its lattice, which is the essential characteristic in the increase in biological performance of HA after implantation. Moreover, the synergistic effect between the trace elements of a natural effect and the incorporation of the doping agent is yet to be investigated due to its capacity to create widely unexplored synergies. Considering the arguments mentioned above, it can be determined that the optimum synthesis process, type of dopant, and concentration still need to be assessed in order to further develop the ideal biomaterial with both superior bioactivity and mechanical strength [205].

Furthermore, biological investigations have not yet shown the efficacy of doped HA, considering in vivo suitability and cytocompatibility. To demonstrate the biological effects, further research, by evaluating the immunoregulation, angiogenesis, osteogenesis, and biomineralization, which are necessary for the osseous tissue regeneration process, is still required.

## 7. Conclusions

HA is the most widely applied ceramic in bone tissue engineering due to its similarity in chemical structure with natural osseous tissue. This material is highly recommended for intrinsic osteoinduction, osteoconduction, and osteogenic properties. Moreover, its high biocompatibility makes it the ideal material to be introduced on implanted sites to enhance bone formation and regeneration. However, there are still some limitations when discussing conventionally synthesized HA, such as its mechanical strength and bioactivity. The perfect solution to solve this issue has been represented by the substitution of the HA structure with doping ions. These doping ions were able to modify the properties of the material and induce some additional effects which encourage the use of doped HA in bone tissue applications. In this review, we have presented the necessity of doping HA, its influence on bioactivity and mechanical properties, and the latest applications of doped materials in biomedical engineering. Furthermore, additional research is still needed to optimize the synthesis methods, the doping concentration, and the influence of these processes on the human body. Moreover, the connection between HA synthesized from natural sources and dopants has not yet been fully investigated.

## Figures and Tables

**Table 1:** The main synthesis methods and properties of synthesized HA.

Synthesis Method	Morphology	Size	Characteristics	References
Sol–gel Method	Spherical, rods, needle, tube	5–1000 nm	Capacity to synthesize homogeneous materials, mechanical properties enhanced due to nanocrystalline grain structures and high purity.	[52,53,54,55]
Hydrothermal	Rod, needle, spherical, rollers, wire, spherulites, leaves, belts	4–140 nm	Low costs, good homogenicity and solubility of the precursors, tailorable nucleation, and growth of the crystal.	[56,57,58]
Chemical Precipitation	Needle, whisker, rods, spherical, platelets	5–130 nm	Low costs, controlled environment, and enhanced porosity.	[39,59,60]
Biosources	Irregular, sphere, flakes, needles, rods	6–180 nm	Capacity to retain trace element, increase bioactivity, and be eco-friendly.	[61,62]
Microemulsion	Spherical, rods, irregular, needle, flakes	5–100 nm	Good anti-aggregation property, outstanding stability, and self-controlled development of apertures.	[63,64,65]
Combustion	Needle, rods, irregular, spherical	40–200 nm	Simplicity, high purity of the obtained products, low energy consumption, and high purity of synthesized material.	[56,66,67]

**Table 2:** Doping material introduced into HA structures and their improved properties [3].

Doping Ion		Biologic Activity
**Cation**	Na^+^	Enhanced osteoconductivity and improved cell proliferation
	K^+^	Enhanced thermal stability
	Mg^2+^	Improved crystal growth, crystallization, thermal stability, and dissolution rate
	Sr^2+^	Enhanced bone regeneration and formation, and inhibited bone resorption
	Mn^2+^	Enhanced cell adhesion
	Zn^2+^	Enhanced bone formation and regeneration, increased antimicrobial activity
**Anion**		
	CO_3_^2−^	High specific surface area, reduced crystallite size, enhanced osteoconductive properties, increased solubility
	SiO_4_^4−^	Enhanced bioactivity, improved dissolution rate
	F^−^	Improved stability, reduced solubility, and increased remineralization
	Cl^−^	Enhanced osteoconductive properties, and increased solubility

## Data Availability

Not applicable.
